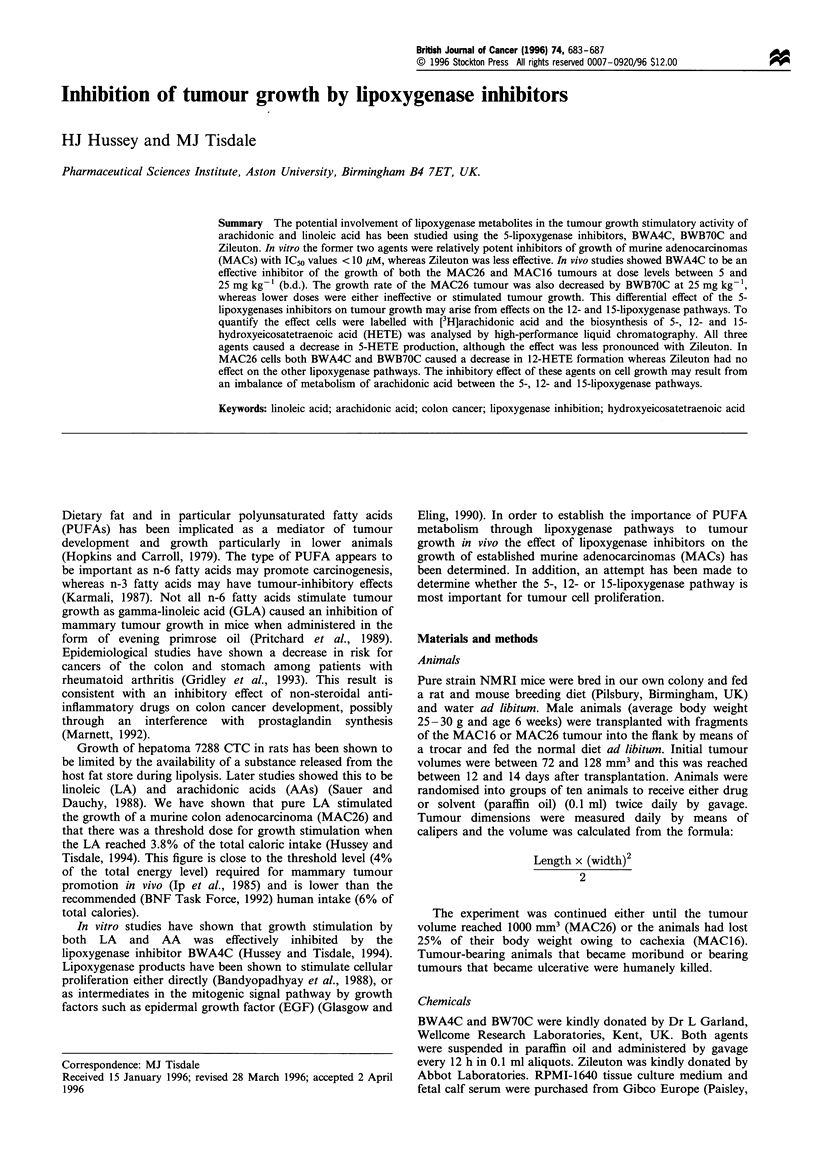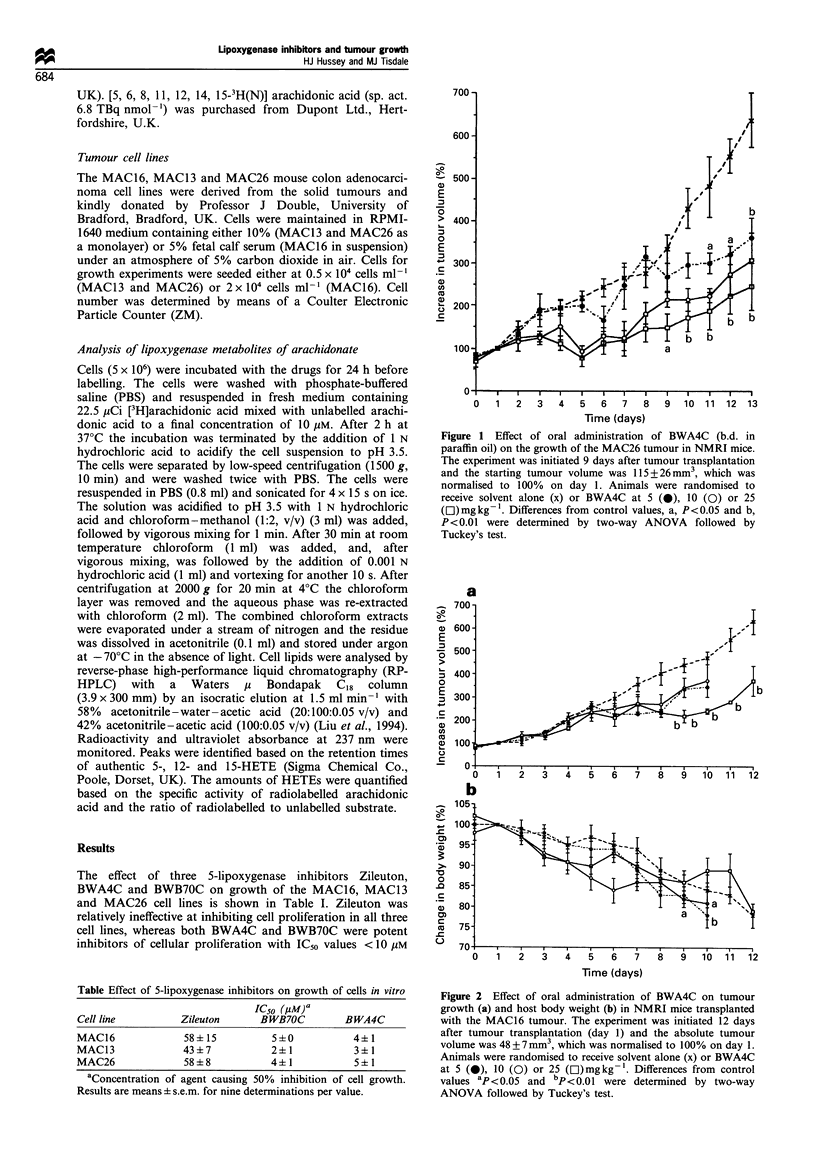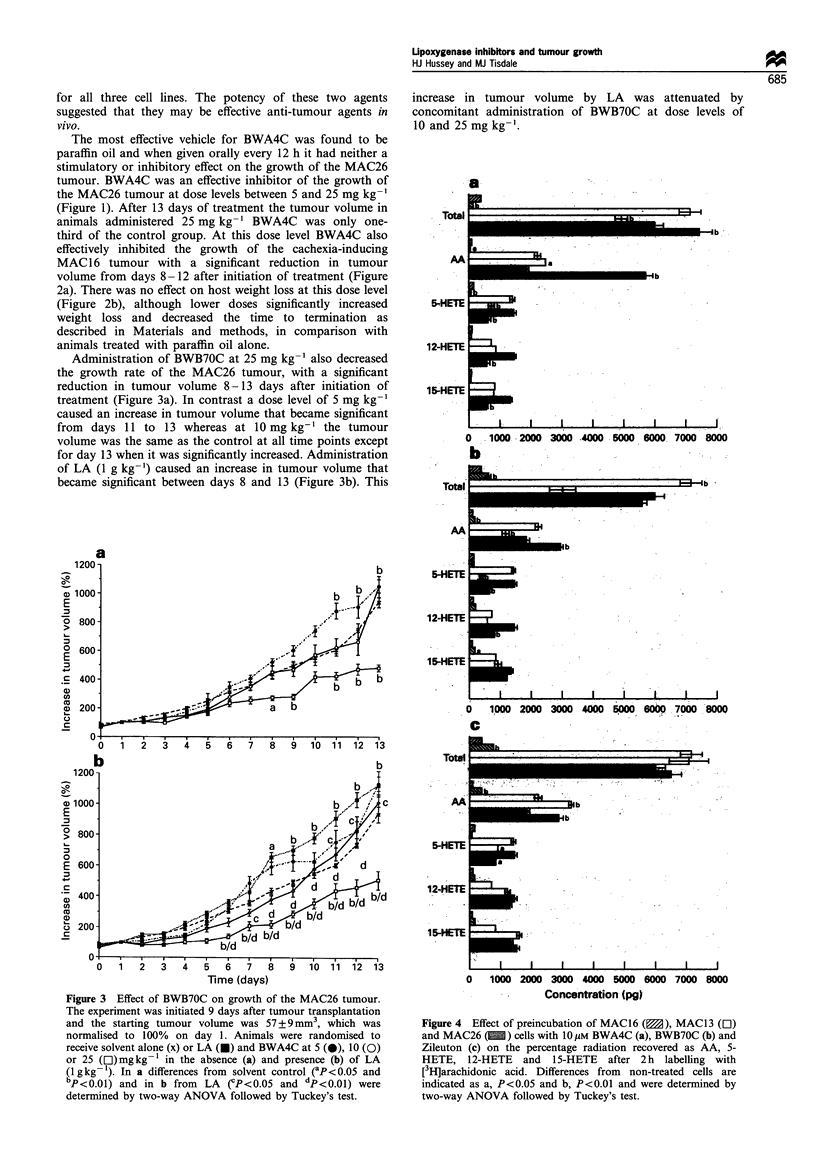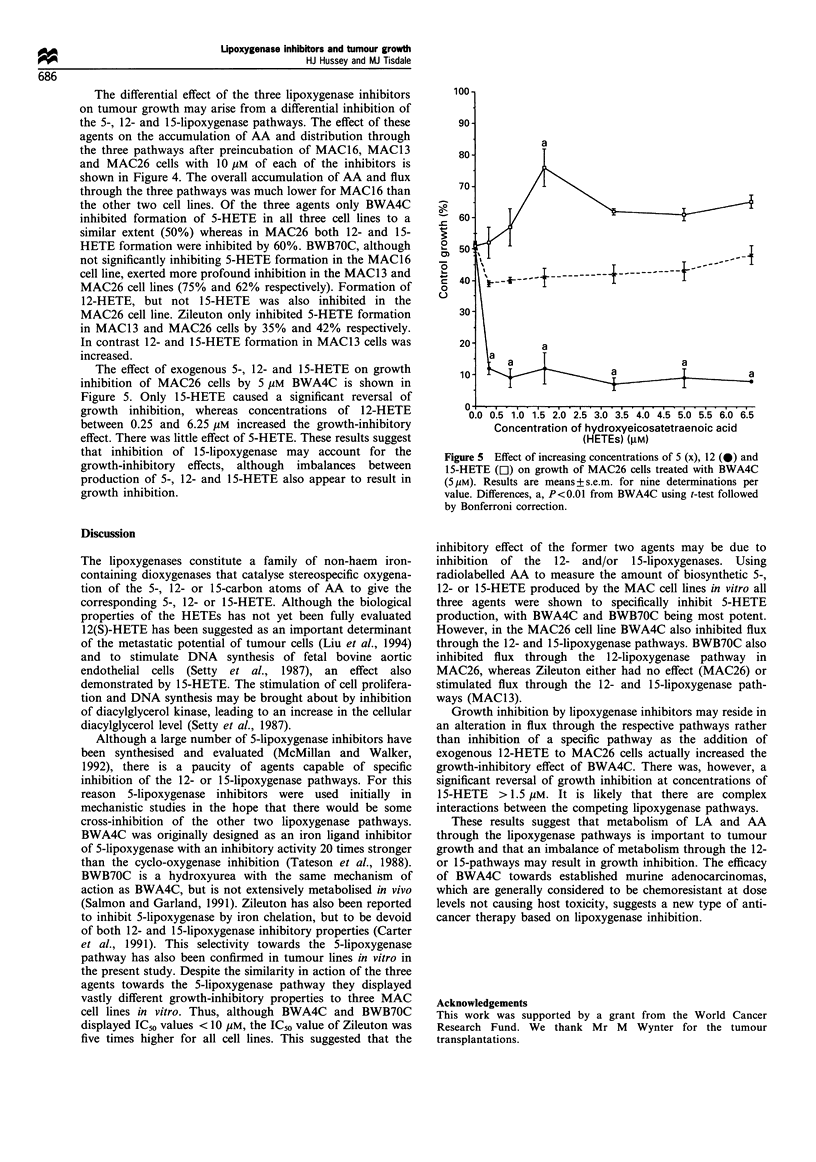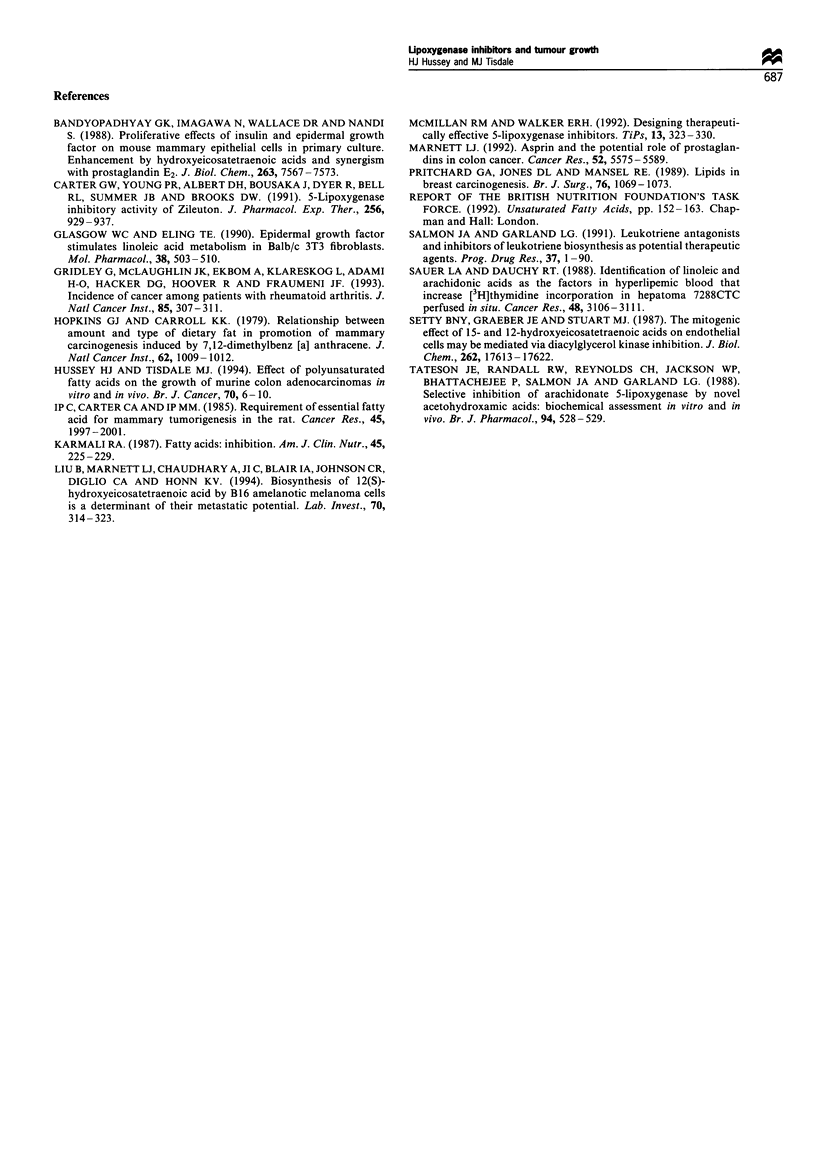# Inhibition of tumour growth by lipoxygenase inhibitors.

**DOI:** 10.1038/bjc.1996.422

**Published:** 1996-09

**Authors:** H. J. Hussey, M. J. Tisdale

**Affiliations:** Pharmaceutical Sciences Institute, Aston University, Birmingham, UK.

## Abstract

The potential involvement of lipoxygenase metabolites in the tumour growth stimulatory activity of arachidonic and linoleic acid has been studied using the 5-lipoxygenase inhibitors, BWA4C, BWB70C and Zileuton. In vitro the former two agents were relatively potent inhibitors of growth of murine adenocarcinomas (MACs) with IC50 values < 10 microM, whereas Zileuton was less effective. In vivo studies showed BWA4C to be an effective inhibitor of the growth of both the MAC26 and MAC16 tumours at dose levels between 5 and 25 mg kg-1 (b.d.). The growth rate of the MAC26 tumour was also decreased by BWB70C at 25 mg kg-1, whereas lower doses were either ineffective or stimulated tumour growth. This differential effect of the 5-lipoxygenases inhibitors on tumour growth may arise from effects on the 12- and 15-lipoxygenase pathways. To quantify the effect cells were labelled with [3H]arachidonic acid and the biosynthesis of 5-, 12- and 15-hydroxyeicosatetraenoic acid (HETE) was analysed by high-performance liquid chromatography. All three agents caused a decrease in 5-HETE production, although the effect was less pronounced with Zileuton. In MAC26 cells both BWA4C and BWB70C caused a decrease in 12-HETE formation whereas Zileuton had no effect on the other lipoxygenase pathways. The inhibitory effect of these agents on cell growth may result from an imbalance of metabolism of arachidonic acid between the 5-, 12- and 15-lipoxygenase pathways.


British Journal of Cancer (1996) 74, 683-687
© 1996 Stockton Press All rights reserved 0007-0920/96 $12.00

### Inhibition of tumour growth by lipoxygenase inhibitors

HJ Hussey and MJ Tisdale

*Pharmaceutical Sciences Institute, Aston University, Birmingham B4 7ET, UK.*

**Summary** The potential involvement of lipoxygenase metabolites in the tumour growth stimulatory activity of arachidonic and linoleic acid has been studied using the 5-lipoxygenase inhibitors, BWA4C, BWB70C and Zileuton. *In vitro* the former two agents were relatively potent inhibitors of growth of murine adenocarcinomas (MACs) with $IC_{50}$ values $< 10$ $\mu M$, whereas Zileuton was less effective. *In vivo* studies showed BWA4C to be an effective inhibitor of the growth of both the MAC26 and MAC16 tumours at dose levels between 5 and 25 mg $kg^{-1}$ (b.d.). The growth rate of the MAC26 tumour was also decreased by BWB70C at 25 mg $kg^{-1}$, whereas lower doses were either ineffective or stimulated tumour growth. This differential effect of the 5-lipoxygenases inhibitors on tumour growth may arise from effects on the 12- and 15-lipoxygenase pathways. To quantify the effect cells were labelled with [$^3$H]arachidonic acid and the biosynthesis of 5-, 12- and 15-hydroxyeicosatetraenoic acid (HETE) was analysed by high-performance liquid chromatography. All three agents caused a decrease in 5-HETE production, although the effect was less pronounced with Zileuton. In MAC26 cells both BWA4C and BWB70C caused a decrease in 12-HETE formation whereas Zileuton had no effect on the other lipoxygenase pathways. The inhibitory effect of these agents on cell growth may result from an imbalance of metabolism of arachidonic acid between the 5-, 12- and 15-lipoxygenase pathways.

**Keywords:** linoleic acid; arachidonic acid; colon cancer; lipoxygenase inhibition; hydroxyeicosatetraenoic acid

Dietary fat and in particular polyunsaturated fatty acids (PUFAs) has been implicated as a mediator of tumour development and growth particularly in lower animals (Hopkins and Carroll, 1979). The type of PUFA appears to be important as n-6 fatty acids may promote carcinogenesis, whereas n-3 fatty acids may have tumour-inhibitory effects (Karmali, 1987). Not all n-6 fatty acids stimulate tumour growth as gamma-linoleic acid (GLA) caused an inhibition of mammary tumour growth in mice when administered in the form of evening primrose oil (Pritchard *et al.*, 1989). Epidemiological studies have shown a decrease in risk for cancers of the colon and stomach among patients with rheumatoid arthritis (Gridley *et al.*, 1993). This result is consistent with an inhibitory effect of non-steroidal anti-inflammatory drugs on colon cancer development, possibly through an interference with prostaglandin synthesis (Marnett, 1992).

Growth of hepatoma 7288 CTC in rats has been shown to be limited by the availability of a substance released from the host fat store during lipolysis. Later studies showed this to be linoleic (LA) and arachidonic acids (AAs) (Sauer and Dauchy, 1988). We have shown that pure LA stimulated the growth of a murine colon adenocarcinoma (MAC26) and that there was a threshold dose for growth stimulation when the LA reached 3.8% of the total caloric intake (Hussey and Tisdale, 1994). This figure is close to the threshold level (4% of the total energy level) required for mammary tumour promotion *in vivo* (Ip *et al.*, 1985) and is lower than the recommended (BNF Task Force, 1992) human intake (6% of total calories).

*In vitro* studies have shown that growth stimulation by both LA and AA was effectively inhibited by the lipoxygenase inhibitor BWA4C (Hussey and Tisdale, 1994). Lipoxygenase products have been shown to stimulate cellular proliferation either directly (Bandyopadhyay *et al.*, 1988), or as intermediates in the mitogenic signal pathway by growth factors such as epidermal growth factor (EGF) (Glasgow and Eling, 1990). In order to establish the importance of PUFA metabolism through lipoxygenase pathways to tumour growth *in vivo* the effect of lipoxygenase inhibitors on the growth of established murine adenocarcinomas (MACs) has been determined. In addition, an attempt has been made to determine whether the 5-, 12- or 15-lipoxygenase pathway is most important for tumour cell proliferation.

Correspondence: MJ Tisdale
Received 15 January 1996; revised 28 March 1996; accepted 2 April 1996

### Materials and methods

### *Animals*

Pure strain NMRI mice were bred in our own colony and fed a rat and mouse breeding diet (Pilsbury, Birmingham, UK) and water *ad libitum.* Male animals (average body weight 25–30 g and age 6 weeks) were transplanted with fragments of the MAC16 or MAC26 tumour into the flank by means of a trocar and fed the normal diet *ad libitum.* Initial tumour volumes were between 72 and 128 mm$^3$ and this was reached between 12 and 14 days after transplantation. Animals were randomised into groups of ten animals to receive either drug or solvent (paraffin oil) (0.1 ml) twice daily by gavage. Tumour dimensions were measured daily by means of calipers and the volume was calculated from the formula:

$$\frac{\text{Length} \times (\text{width})^2}{2}$$

The experiment was continued either until the tumour volume reached 1000 mm$^3$ (MAC26) or the animals had lost 25% of their body weight owing to cachexia (MAC16). Tumour-bearing animals that became moribund or bearing tumours that became ulcerative were humanely killed.

### *Chemicals*

BWA4C and BW70C were kindly donated by Dr L Garland, Wellcome Research Laboratories, Kent, UK. Both agents were suspended in paraffin oil and administered by gavage every 12 h in 0.1 ml aliquots. Zileuton was kindly donated by Abbot Laboratories. RPMI-1640 tissue culture medium and fetal calf serum were purchased from Gibco Europe (Paisley,

UK). [5, 6, 8, 11, 12, 14, 15-$^3$H(N)] arachidonic acid (sp. act. 6.8 TBq nmol$^{-1}$) was purchased from Dupont Ltd., Hertfordshire, U.K.

*Tumour cell lines*

The MAC16, MAC13 and MAC26 mouse colon adenocarcinoma cell lines were derived from the solid tumours and kindly donated by Professor J Double, University of Bradford, Bradford, UK. Cells were maintained in RPMI-1640 medium containing either 10% (MAC13 and MAC26 as a monolayer) or 5% fetal calf serum (MAC16 in suspension) under an atmosphere of 5% carbon dioxide in air. Cells for growth experiments were seeded either at $0.5 \times 10^4$ cells ml$^{-1}$ (MAC13 and MAC26) or $2 \times 10^4$ cells ml$^{-1}$ (MAC16). Cell number was determined by means of a Coulter Electronic Particle Counter (ZM).

*Analysis of lipoxygenase metabolites of arachidonate*

Cells ($5 \times 10^6$) were incubated with the drugs for 24 h before labelling. The cells were washed with phosphate-buffered saline (PBS) and resuspended in fresh medium containing 22.5 $\mu$Ci [$^3$H]arachidonic acid mixed with unlabelled arachidonic acid to a final concentration of 10 $\mu$M. After 2 h at 37°C the incubation was terminated by the addition of 1 N hydrochloric acid to acidify the cell suspension to pH 3.5. The cells were separated by low-speed centrifugation (1500 *g*, 10 min) and were washed twice with PBS. The cells were resuspended in PBS (0.8 ml) and sonicated for $4 \times 15$ s on ice. The solution was acidified to pH 3.5 with 1 N hydrochloric acid and chloroform–methanol (1:2, v/v) (3 ml) was added, followed by vigorous mixing for 1 min. After 30 min at room temperature chloroform (1 ml) was added, and, after vigorous mixing, was followed by the addition of 0.001 N hydrochloric acid (1 ml) and vortexing for another 10 s. After centrifugation at 2000 *g* for 20 min at 4°C the chloroform layer was removed and the aqueous phase was re-extracted with chloroform (2 ml). The combined chloroform extracts were evaporated under a stream of nitrogen and the residue was dissolved in acetonitrile (0.1 ml) and stored under argon at −70°C in the absence of light. Cell lipids were analysed by reverse-phase high-performance liquid chromatography (RP-HPLC) with a Waters $\mu$ Bondapak $C_{18}$ column ($3.9 \times 300$ mm) by an isocratic elution at 1.5 ml min$^{-1}$ with 58% acetonitrile–water–acetic acid (20:100:0.05 v/v) and 42% acetonitrile–acetic acid (100:0.05 v/v) (Liu *et al.*, 1994). Radioactivity and ultraviolet absorbance at 237 nm were monitored. Peaks were identified based on the retention times of authentic 5-, 12- and 15-HETE (Sigma Chemical Co., Poole, Dorset, UK). The amounts of HETEs were quantified based on the specific activity of radiolabelled arachidonic acid and the ratio of radiolabelled to unlabelled substrate.

### Results

The effect of three 5-lipoxygenase inhibitors Zileuton, BWA4C and BWB70C on growth of the MAC16, MAC13 and MAC26 cell lines is shown in Table I. Zileuton was relatively ineffective at inhibiting cell proliferation in all three cell lines, whereas both BWA4C and BWB70C were potent inhibitors of cellular proliferation with $IC_{50}$ values $<10$ $\mu$M

**Table** Effect of 5-lipoxygenase inhibitors on growth of cells *in vitro*

| *Cell line* | *Zileuton* | *$IC_{50}$ ($\mu$M)$^a$* *BWB70C* | *BWA4C* |
|---|---|---|---|
| MAC16 | 58±15 | 5±0 | 4±1 |
| MAC13 | 43±7 | 2±1 | 3±1 |
| MAC26 | 58±8 | 4±1 | 5±1 |

$^a$Concentration of agent causing 50% inhibition of cell growth. Results are means ± s.e.m. for nine determinations per value.

**Figure 1** Effect of oral administration of BWA4C (b.d. in paraffin oil) on the growth of the MAC26 tumour in NMRI mice. The experiment was initiated 9 days after tumour transplantation and the starting tumour volume was $115 \pm 26$ mm$^3$, which was normalised to 100% on day 1. Animals were randomised to receive solvent alone (x) or BWA4C at 5 (●), 10 (○) or 25 (□) mg kg$^{-1}$. Differences from control values, a, $P<0.05$ and b, $P<0.01$ were determined by two-way ANOVA followed by Tuckey's test.

**Figure 2** Effect of oral administration of BWA4C on tumour growth (**a**) and host body weight (**b**) in NMRI mice transplanted with the MAC16 tumour. The experiment was initiated 12 days after tumour transplantation (day 1) and the absolute tumour volume was $48 \pm 7$ mm$^3$, which was normalised to 100% on day 1. Animals were randomised to receive solvent alone (x) or BWA4C at 5 (●), 10 (○) or 25 (□) mg kg$^{-1}$. Differences from control values $^aP<0.05$ and $^bP<0.01$ were determined by two-way ANOVA followed by Tuckey's test.

for all three cell lines. The potency of these two agents suggested that they may be effective anti-tumour agents *in vivo*.

The most effective vehicle for BWA4C was found to be paraffin oil and when given orally every 12 h it had neither a stimulatory or inhibitory effect on the growth of the MAC26 tumour. BWA4C was an effective inhibitor of the growth of the MAC26 tumour at dose levels between 5 and 25 mg $kg^{-1}$ (Figure 1). After 13 days of treatment the tumour volume in animals administered 25 mg $kg^{-1}$ BWA4C was only one-third of the control group. At this dose level BWA4C also effectively inhibited the growth of the cachexia-inducing MAC16 tumour with a significant reduction in tumour volume from days 8–12 after initiation of treatment (Figure 2a). There was no effect on host weight loss at this dose level (Figure 2b), although lower doses significantly increased weight loss and decreased the time to termination as described in Materials and methods, in comparison with animals treated with paraffin oil alone.

Administration of BWB70C at 25 mg $kg^{-1}$ also decreased the growth rate of the MAC26 tumour, with a significant reduction in tumour volume 8–13 days after initiation of treatment (Figure 3a). In contrast a dose level of 5 mg $kg^{-1}$ caused an increase in tumour volume that became significant from days 11 to 13 whereas at 10 mg $kg^{-1}$ the tumour volume was the same as the control at all time points except for day 13 when it was significantly increased. Administration of LA (1 g $kg^{-1}$) caused an increase in tumour volume that became significant between days 8 and 13 (Figure 3b). This increase in tumour volume by LA was attenuated by concomitant administration of BWB70C at dose levels of 10 and 25 mg $kg^{-1}$.

**Figure 3** Effect of BWB70C on growth of the MAC26 tumour. The experiment was initiated 9 days after tumour transplantation and the starting tumour volume was $57 \pm 9 mm^3$, which was normalised to 100% on day 1. Animals were randomised to receive solvent alone (x) or LA (■) and BWA4C at 5 (●), 10 (○) or 25 (□) mg $kg^{-1}$ in the absence (**a**) and presence (**b**) of LA (1 g $kg^{-1}$). In **a** differences from solvent control ($^aP<0.05$ and $^bP<0.01$) and in **b** from LA ($^cP<0.05$ and $^dP<0.01$) were determined by two-way ANOVA followed by Tuckey's test.

**Figure 4** Effect of preincubation of MAC16 (▨), MAC13 (□) and MAC26 (▩) cells with 10 $\mu$M BWA4C (**a**), BWB70C (**b**) and Zileuton (**c**) on the percentage radiation recovered as AA, 5-HETE, 12-HETE and 15-HETE after 2 h labelling with [$^3$H]arachidonic acid. Differences from non-treated cells are indicated as a, $P<0.05$ and b, $P<0.01$ and were determined by two-way ANOVA followed by Tuckey's test.

The differential effect of the three lipoxygenase inhibitors on tumour growth may arise from a differential inhibition of the 5-, 12- and 15-lipoxygenase pathways. The effect of these agents on the accumulation of AA and distribution through the three pathways after preincubation of MAC16, MAC13 and MAC26 cells with 10 $\mu$M of each of the inhibitors is shown in Figure 4. The overall accumulation of AA and flux through the three pathways was much lower for MAC16 than the other two cell lines. Of the three agents only BWA4C inhibited formation of 5-HETE in all three cell lines to a similar extent (50%) whereas in MAC26 both 12- and 15-HETE formation were inhibited by 60%. BWB70C, although not significantly inhibiting 5-HETE formation in the MAC16 cell line, exerted more profound inhibition in the MAC13 and MAC26 cell lines (75% and 62% respectively). Formation of 12-HETE, but not 15-HETE was also inhibited in the MAC26 cell line. Zileuton only inhibited 5-HETE formation in MAC13 and MAC26 cells by 35% and 42% respectively. In contrast 12- and 15-HETE formation in MAC13 cells was increased.

The effect of exogenous 5-, 12- and 15-HETE on growth inhibition of MAC26 cells by 5 $\mu$M BWA4C is shown in Figure 5. Only 15-HETE caused a significant reversal of growth inhibition, whereas concentrations of 12-HETE between 0.25 and 6.25 $\mu$M increased the growth-inhibitory effect. There was little effect of 5-HETE. These results suggest that inhibition of 15-lipoxygenase may account for the growth-inhibitory effects, although imbalances between production of 5-, 12- and 15-HETE also appear to result in growth inhibition.

**Figure 5** Effect of increasing concentrations of 5 (x), 12 (●) and 15-HETE (□) on growth of MAC26 cells treated with BWA4C (5 $\mu$M). Results are means ± s.e.m. for nine determinations per value. Differences, a, $P<0.01$ from BWA4C using *t*-test followed by Bonferroni correction.

### Discussion

The lipoxygenases constitute a family of non-haem iron-containing dioxygenases that catalyse stereospecific oxygenation of the 5-, 12- or 15-carbon atoms of AA to give the corresponding 5-, 12- or 15-HETE. Although the biological properties of the HETEs has not yet been fully evaluated 12(S)-HETE has been suggested as an important determinant of the metastatic potential of tumour cells (Liu *et al.*, 1994) and to stimulate DNA synthesis of fetal bovine aortic endothelial cells (Setty *et al.*, 1987), an effect also demonstrated by 15-HETE. The stimulation of cell proliferation and DNA synthesis may be brought about by inhibition of diacylglycerol kinase, leading to an increase in the cellular diacylglycerol level (Setty *et al.*, 1987).

Although a large number of 5-lipoxygenase inhibitors have been synthesised and evaluated (McMillan and Walker, 1992), there is a paucity of agents capable of specific inhibition of the 12- or 15-lipoxygenase pathways. For this reason 5-lipoxygenase inhibitors were used initially in mechanistic studies in the hope that there would be some cross-inhibition of the other two lipoxygenase pathways. BWA4C was originally designed as an iron ligand inhibitor of 5-lipoxygenase with an inhibitory activity 20 times stronger than the cyclo-oxygenase inhibition (Tateson *et al.*, 1988). BWB70C is a hydroxyurea with the same mechanism of action as BWA4C, but is not extensively metabolised *in vivo* (Salmon and Garland, 1991). Zileuton has also been reported to inhibit 5-lipoxygenase by iron chelation, but to be devoid of both 12- and 15-lipoxygenase inhibitory properties (Carter *et al.*, 1991). This selectivity towards the 5-lipoxygenase pathway has also been confirmed in tumour lines *in vitro* in the present study. Despite the similarity in action of the three agents towards the 5-lipoxygenase pathway they displayed vastly different growth-inhibitory properties to three MAC cell lines *in vitro*. Thus, although BWA4C and BWB70C displayed $IC_{50}$ values $<10$ $\mu$M, the $IC_{50}$ value of Zileuton was five times higher for all cell lines. This suggested that the inhibitory effect of the former two agents may be due to inhibition of the 12- and/or 15-lipoxygenases. Using radiolabelled AA to measure the amount of biosynthetic 5-, 12- or 15-HETE produced by the MAC cell lines *in vitro* all three agents were shown to specifically inhibit 5-HETE production, with BWA4C and BWB70C being most potent. However, in the MAC26 cell line BWA4C also inhibited flux through the 12- and 15-lipoxygenase pathways. BWB70C also inhibited flux through the 12-lipoxygenase pathway in MAC26, whereas Zileuton either had no effect (MAC26) or stimulated flux through the 12- and 15-lipoxygenase pathways (MAC13).

Growth inhibition by lipoxygenase inhibitors may reside in an alteration in flux through the respective pathways rather than inhibition of a specific pathway as the addition of exogenous 12-HETE to MAC26 cells actually increased the growth-inhibitory effect of BWA4C. There was, however, a significant reversal of growth inhibition at concentrations of 15-HETE $>1.5$ $\mu$M. It is likely that there are complex interactions between the competing lipoxygenase pathways.

These results suggest that metabolism of LA and AA through the lipoxygenase pathways is important to tumour growth and that an imbalance of metabolism through the 12- or 15-pathways may result in growth inhibition. The efficacy of BWA4C towards established murine adenocarcinomas, which are generally considered to be chemoresistant at dose levels not causing host toxicity, suggests a new type of anti-cancer therapy based on lipoxygenase inhibition.

**Acknowledgements**
This work was supported by a grant from the World Cancer Research Fund. We thank Mr M Wynter for the tumour transplantations.

### References

BANDYOPADHYAY GK, IMAGAWA N, WALLACE DR AND NANDI S. (1988). Proliferative effects of insulin and epidermal growth factor on mouse mammary epithelial cells in primary culture. Enhancement by hydroxyeicosatetraenoic acids and synergism with prostaglandin $E_2$. *J. Biol. Chem.*, **263**, 7567–7573.

CARTER GW, YOUNG PR, ALBERT DH, BOUSAKA J, DYER R, BELL RL, SUMMER JB AND BROOKS DW. (1991). 5-Lipoxygenase inhibitory activity of Zileuton. *J. Pharmacol. Exp. Ther.*, **256**, 929–937.

GLASGOW WC AND ELING TE. (1990). Epidermal growth factor stimulates linoleic acid metabolism in Balb/c 3T3 fibroblasts. *Mol. Pharmacol.*, **38**, 503–510.

GRIDLEY G, MCLAUGHLIN JK, EKBOM A, KLARESKOG L, ADAMI H-O, HACKER DG, HOOVER R AND FRAUMENI JF. (1993). Incidence of cancer among patients with rheumatoid arthritis. *J. Natl Cancer Inst.*, **85**, 307–311.

HOPKINS GJ AND CARROLL KK. (1979). Relationship between amount and type of dietary fat in promotion of mammary carcinogenesis induced by 7,12-dimethylbenz [a] anthracene. *J. Natl Cancer Inst.*, **62**, 1009–1012.

HUSSEY HJ AND TISDALE MJ. (1994). Effect of polyunsaturated fatty acids on the growth of murine colon adenocarcinomas *in vitro* and *in vivo*. *Br. J. Cancer*, **70**, 6–10.

IP C, CARTER CA AND IP MM. (1985). Requirement of essential fatty acid for mammary tumorigenesis in the rat. *Cancer Res.*, **45**, 1997–2001.

KARMALI RA. (1987). Fatty acids: inhibition. *Am. J. Clin. Nutr.*, **45**, 225–229.

LIU B, MARNETT LJ, CHAUDHARY A, JI C, BLAIR IA, JOHNSON CR, DIGLIO CA AND HONN KV. (1994). Biosynthesis of 12(S)-hydroxyeicosatetraenoic acid by B16 amelanotic melanoma cells is a determinant of their metastatic potential. *Lab. Invest.*, **70**, 314–323.

MCMILLAN RM AND WALKER ERH. (1992). Designing therapeutically effective 5-lipoxygenase inhibitors. *TiPs*, **13**, 323–330.

MARNETT LJ. (1992). Asprin and the potential role of prostaglandins in colon cancer. *Cancer Res.*, **52**, 5575–5589.

PRITCHARD GA, JONES DL AND MANSEL RE. (1989). Lipids in breast carcinogenesis. *Br. J. Surg.*, **76**, 1069–1073.

REPORT OF THE BRITISH NUTRITION FOUNDATION'S TASK FORCE. (1992). *Unsaturated Fatty Acids*, pp. 152–163. Chapman and Hall: London.

SALMON JA AND GARLAND LG. (1991). Leukotriene antagonists and inhibitors of leukotriene biosynthesis as potential therapeutic agents. *Prog. Drug Res.*, **37**, 1–90.

SAUER LA AND DAUCHY RT. (1988). Identification of linoleic and arachidonic acids as the factors in hyperlipemic blood that increase [$^3$H]thymidine incorporation in hepatoma 7288CTC perfused *in situ*. *Cancer Res.*, **48**, 3106–3111.

SETTY BNY, GRAEBER JE AND STUART MJ. (1987). The mitogenic effect of 15- and 12-hydroxyeicosatetraenoic acids on endothelial cells may be mediated via diacylglycerol kinase inhibition. *J. Biol. Chem.*, **262**, 17613–17622.

TATESON JE, RANDALL RW, REYNOLDS CH, JACKSON WP, BHATTACHEJEE P, SALMON JA AND GARLAND LG. (1988). Selective inhibition of arachidonate 5-lipoxygenase by novel acetohydroxamic acids: biochemical assessment *in vitro* and *in vivo*. *Br. J. Pharmacol.*, **94**, 528–529.